# The Hydropathy Index of the HCDR3 Region of the B-Cell Receptor Identifies Two Subgroups of IGHV-Mutated Chronic Lymphocytic Leukemia Patients With Distinct Outcome

**DOI:** 10.3389/fonc.2021.723722

**Published:** 2021-10-26

**Authors:** Arancha Rodríguez-Caballero, Blanca Fuentes Herrero, Guillermo Oliva Ariza, Ignacio Criado, Miguel Alcoceba, Carlos Prieto, María Pérez Caro, Andrés C. García-Montero, Marcos González Díaz, Francesco Forconi, Ana Bela Sarmento-Ribeiro, Julia Almeida, Alberto Orfao

**Affiliations:** ^1^ Translational and Clinical Research Program, Cancer Research Center Institute of Cancer Molecular and Cellular Biology (IBMCC), University of Salamanca-The Spanish National Research Council (USAL-CSIC), Department of Medicine and Cytometry Service, Nucleus Research Support Platform from University of Salamanca (NUCLEUS), University of Salamanca, Salamanca, Spain; ^2^ CIBERONC Program of Liquid Biopsy, Hematologic Tumors, Centro de Investigación Biomédica en Red de Cáncer CB16/12/00400 and CB16/12/00233 (CIBERONC), Madrid, Spain; ^3^ Molecular and Cellular Biology of Hematologic Tumors, Biomedical Research Institute of Salamanca (IBSAL), Salamanca, Spain; ^4^ Department of Hematology, University Hospital of Salamanca/Biomedical Research Institute of Salamanca (HUS/IBSAL), Salamanca, Spain; ^5^ Bioinformatics Service, Nucleus Research Support Platform from University of Salamanca (NUCLEUS), University of Salamanca, Salamanca, Spain; ^6^ Spanish National DNA Bank Carlos III, University of Salamanca, Salamanca, Spain; ^7^ Haematology Oncology Group, School of Cancer Sciences, Cancer Research UK Centre and National Institute for Health Research Experimental Cancer Medicine, University of Southampton, Faculty of Medicine, Southampton, United Kingdom; ^8^ Faculty of Medicine, University of Coimbra (FMUC), Coimbra, Portugal; ^9^ Centro Hospitalar e Universitário de Coimbra (CHUC), Coimbra, Portugal

**Keywords:** hydropathy index, neutral HCDR3, negatively charged HCDR3, mutated CLL (M-CLL), disease progression

## Abstract

The HCDR3 sequences of the B-cell receptor (BCR) undergo constraints in length, amino acid use, and charge during maturation of B-cell precursors and after antigen encounter, leading to BCR and antibodies with high affinity to specific antigens. Chronic lymphocytic leukemia consists of an expansion of B-cells with a mixed immature and “antigen-experienced” phenotype, with either a mutated (M-CLL) or unmutated (U-CLL) tumor BCR, associated with distinct patient outcomes. Here, we investigated the hydropathy index of the BCR of 138 CLL patients and its association with the IGHV mutational status and patient outcome. Overall, two clearly distinct subgroups of M-CLL patients emerged, based on a neutral (mean hydropathy index of -0.1) vs. negatively charged BCR (mean hydropathy index of -1.1) with molecular features closer to those of B-cell precursors and peripheral/mature B-cells, respectively. Despite that M-CLL with neutral HCDR3 did not show traits associated with a mature B-cell repertoire, important differences in IGHV gene usage of tumor cells and patient outcome were observed in this subgroup of patients once compared to both U-CLL and M-CLL with negatively charged HCDR3 sequences. Compared to M-CLL with negatively charged HCDR3 sequences, M-CLL with neutral HCDR3 sequences showed predominance of men, more advanced stages of the disease, and a greater frequency of genetic alterations—e.g., del(17p)—together with a higher rate of disease progression and shorter time to therapy (TTT), independently of other prognostic factors. Our data suggest that the hydropathy index of the HCDR3 sequences of CLL cells allows the identification of a subgroup of M-CLL with intermediate prognostic features between U-CLL and the more favorable subgroup of M-CLL with a negatively charged BCR.

## Introduction

Chronic lymphocytic leukemia (CLL) is the most prevalent leukemia in adults in the Western world, which is characterized by an expansion of mature-appearing CD5^+^CD20^lo^ B-cells showing an antigen-experienced CD27^+^, IgM^+^, and/or IgD^+^ unswitched phenotype, in association with either an unmutated (U-CLL) or mutated (M-CLL) B-cell receptor (BCR) (1). At diagnosis, most CLL patients show stable disease with a variable number of tumor B-cells in blood (always >5,000 cells/μl) and bone marrow (BM), in the absence of organomegalies, and they do not require active therapy (2). Despite this, a significant fraction of patients shows more advanced disease already at diagnosis or they experience disease progression during follow-up, which translates into the need for active cytotoxic therapy (3).

In the last decades, the mutational status of the immunoglobulin (IG) heavy-chain variable (IGHV) genes that code for the BCR, together with disease stage and tumor cytogenetics, has emerged among other variables, as relevant prognostic factors in CLL (4). Thus, U-CLL patients show a significantly poorer outcome compared to M-CLL (4). Thereby, analysis of the IGHV status is currently part of the core variables investigated in the diagnostic workup of this disease (3, 5). Despite this, M-CLL patients have a heterogeneous outcome (6, 7).

From a pathogenic point of view, U-CLL cells resemble “pre-germinal center” (pre-GC) B-cells, whereas M-CLL cells mimic “post-GC” B-lymphocytes (8, 9). However, tumor cells from both CLL groups typically display a mixed immature (CD5^+^ CD23^+^) and “antigen-experienced” (CD27^+^) B-cell phenotype (10), suggesting they might represent the leukemic counterpart of B-lymphocytes that might have undergone BCR stimulation in the GC (M-CLL) vs. peripheral tissues, following selection of B-cell precursors in BM (U-CLL). In line with this hypothesis, the IGHV1-69/IGHJ6 genes which show highly similar junctional regions to those of normal peripheral blood (PB) CD5^+^ GC B-cells are more frequently represented among U-CLL, supporting a close relationship between U-CLL cells and the B-cells responsible for the natural antibody repertoire (11). This potential relationship is further supported by the fact that most normal CD5^+^ B-cells isolated from blood correspond to immature and (early) naïve B-cells that express unmutated VH gene regions (12). In turn, B-cell activation *via* T-cell-dependent antigens leads to the expansion of hypermutated germinal center (GC)-derived B-cells (13), suggesting that M-CLL might be associated with the “classical unswitched memory B-cell” compartment, despite that some M-CLL also show BCR features that overlap with those of natural antibodies (14).

Another important biological feature of CLL is the usage of a biased IGHV-D-J repertoire (the so-called “stereotyped” BCR) (9) in around one-third of cases, particularly in U-CLL patients, with important pathogenic and prognostic implications (15, 16). In contrast to U-CLL, the higher load of somatic mutations in the BCR of M-CLL cases makes recognition of common amino acid (aa) patterns in the HCDR3 region more difficult (17). However, other HCDR3 characteristics, such as its overall charge and hydropathy index, might also contribute to better understand the ontogeny of tumor B-cells in CLL, the affinity and specificity profile of their BCR, and its relationship with antigen-driven B-cell responses, even at earlier stages of B-cell maturation (18). In fact, the HCDR3 sequence of the BCR undergoes constraints in length, amino acid use, and charge along the B-cell development and maturation (19). Consequently, the BCR repertoire of early B-cell progenitors is first focused into what appears to be a preferred range for functional antigen recognition by mature B-cells, and subsequently modified after antigen recognition, in order to generate high-affinity antigen-specific antibodies and memory B-cells (19, 20). Interestingly, receptor prototypes based on HCDR3 charge and its association with certain V gene characteristics have been defined in CLL cells with the possibility that such receptor restrictions could reflect selections of the BCR repertoire that have occurred among both antigen-experienced and naive B cells (21). Despite this, the hydropathy features of HCDR3 and its association with the IGHV mutational status and other clinical and biological features of the disease have not been systematically explored in large series of CLL and related with patient outcome.

Here we investigated the hydropathy index of the BCR of tumor cells from 138 CLL patients, and its potential association with other features of the disease, including the BCR mutational status and patient outcome.

## Materials and Methods

### Patients and Samples

A total of 138 untreated CLL patients—81 males and 57 females; median age (range) at diagnosis of 63 years (y) (33–84 y)—diagnosed at the University Hospital of Salamanca (Salamanca, Spain) were studied. Most cases (95/138) had Binet stage A CLL, and 43 had more advanced CLL (Binet B, 22; and Binet C, 21 patients). Median follow-up at the time of study closure was 8 y; at that time, 65 patients (47%) had progressed and required therapy and 24 (17%) had died (**Table 1**). In every patient, genomic DNA (gDNA) from purified CLL B-cells was obtained for molecular investigations. The study was approved by the local institutional Ethics Committee (approval code: CEIC-PI4705/2017). All patients gave their written informed consent to participate to the study in agreement with the Declaration of Helsinki.

**Table 1:** Clinical and biological features of CLL patients classified according to the HCDR3 hydropathy index.

Patient features	CLL with neutral HCDR3 (N = 65)	CLL with negatively charged HCDR3 (N = 73)	*p-value*
Age at diagnosis (years)	63 (33–84)	65 (35–84)	0.47
Men/women^#^	47/18 (72%/28%)	34/39 (47%/53%)	0.002
Binet stage^#^			
A	37 (57%)	58 (79%)	0.006
B	12 (18%)	10 (14%)	
C	16 (25%)	5 (7%)	
Rai stage^#^			
0	30/65 (46%)	54/73 (74%)	0.01
I	6/65 (9%)	7/73 (10%)	
II	9/65 (14%)	5/73 (7%)	
III	6/65 (9%)	2/73 (3%)	
IV	14/65 (21%)	5/73 (7%)	
Hemoglobin (g/L)	140 (70–180)	130 (90–170)	0.94
Anemia (<100 g of hemoglobin/L)^#^	7/65 (11%)	3/71 (4%)	0.13
N. of platelets (×10^9^/L)	154 (15–429)	157 (48–448)	0.11
Thrombocytopenia (<100 × 10^9^ platelets/L)^#^	14/65 (21%)	5/70 (7%)	0.01
N. of PB leukocytes (×10^9^/L)	36 (6–352)	34 (8–576)	0.40
N. of PB total T-cells (×10^9^/L)	3.2 (0.8–7.4)	2.8 (0.5–14)	0.68
N. of PB CD4^+^ T-cells (×10^9^/L)	1.6 (0.5–4.4)	1.7 (0.3–5.9)	0.64
N. of PB CD8^+^ T-cells (×10^9^/L)	0.8 (0.1–3.5)	0.9 (0.1–8)	0.60
N. of PB monocytes (×10^9^/L)	0.6 (0.01–2)	0.4 (0.01–2.9)	0.24
N. of PB neutrophils (×10^9^/L)	6.4 (0.9–20.7)	6.1 (0.2–19.4)	0.50
N. of PB basophils (×10^9^/L)	0.07 (0.005–0.5)	0.06 (0.015–0.6)	1.0
N. of PB eosinophils (×10^9^/L)	0.2 (0.02–1.7)	0.2 (0.01–1.2)	0.16
N. of PB NK cells (×10^9^/L)	0.6 (0.04–2.1)	0.5 (0.04–3.2)	0.60
Tumor B-cell clone size in blood (×10^9^/L)	41.4 (1.4–334.8)	20.9 (0.8–278.9)	0.11
U-CLL/M-CLL^#^	35 (54%)/30 (46%)	25 (34%)/48 (66%)	0.02
% IGHV homology with germ line counterpart HCDR3 length of CLL clone (N. of aa)	99% (86%–100%) 19 (8–28)	96% (85%–100%) 16 (7–25)	0.04 0.004
Cytogenetically altered CLL patients^#^	55/65 (85%)	59/72 (82%)	0.43
Del(13q14)(*D13S25*)^#^	22/65 (34%)	32/72 (44%)	0.14
Trisomy12^#^	16/65 (25%)	11/72 (15%)	0.12
Del(11q)(*ATM*)^#^	6/65 (9%)	6/72 (10%)	0.54
Del(17p)^#^	3/62 (5%)	1/69 (1%)	0.29
N. of cytogenetically altered CLL cells (×10^9^/L)	19 (0–233)	11 (0–185)	0.19
Disease progression^#^	39/65 (60%)	26/73 (36%)	0.003
TTT < 2 years^#^	20/65 (31%)	8/73 (11%)	0.004
Deaths^#^	11/65 (17%)	13/73 (18%)	0.54

Results expressed as median (range) or as # number of cases (percentage).

aa, amino acids; CLL, chronic lymphocytic leukemia; M, mutated IGHV; N, number; PB, peripheral blood; TTT, time to therapy; U, unmutated IGHV.

### *IGHV-D-J* Gene Rearrangement Studies

Analysis of the tumor *IGHV-D-J* gene rearrangements was performed by polymerase chain reaction (PCR) of gDNA from fluorescence-activated cell sorting (FACS)-purified tumor CLL cells according to the ERIC protocols (22), as previously described in detail (23, 24). For *IGHV* sequencing, PCR amplicons were subjected to direct sequencing on both strands. Sequence data were analyzed using the IMGT databases and the IMGT/V-QUEST tool (http://www.imgt.org). Classification into the U-CLL vs. M-CLL categories was based on the well-established 98% cutoff identity to the germline sequence (U-CLL: 98%–100%; M-CLL: <98%) (21).

### Calculation of the Hydropathy Index of HCDR3 Protein Sequences

To determine the hydropathy index—grand average of hydropathy (GRAVY) score—of the HCDR3 protein sequences, the ProtScale Tool from the ExPASy Bioinformatics Resource Portal (https://web.expasy.org/protscale/) and the amino acid (aa) scale values, as defined by Kyte and Doolittle (25), were used (**Supplementary Figures 1A, B**). The Gravy score (GS) was calculated for each HCDR3 sequence by summing up the hydropathy index value of each amino acid residue in the individual HCDR3 sequences and dividing the sum obtained by the number of amino acids in each specific sequence (23) (**Supplementary Table 1** and **Supplementary Figures 1B, C**). Since the HCDR3 hydropathy index in humans follows a Gaussian distribution centered in the neutral/hydrophilic range (average charge: -0.5) (19), each HCDR3 sequence was classified into the neutral HCDR3 (GS ≥ -0.5) or negatively charged HCDR3 (GS < -0.5) categories.

### Cytogenetic Analyses

The most common cytogenetic alterations associated with CLL—i.e., del(13q14), trisomy 12, del(11q) (*ATM*), and del(17p) (*TP53*)—were investigated by iFISH on *FACS*-purified (single) tumor B cells (≥95% purity), as described elsewhere (23, 26).

### Statistical Methods

For all continuous variables, median (range) values were calculated, while for categorical variables, frequencies were used. Either the Mann–Whitney U test or the chi-squared test was used to establish the statistical significance of differences observed between two groups, for continuous and categorical variables, respectively. To avoid associations occurring by chance due to multiple simultaneous comparisons, p-values were Bonferroni-adjusted for comparisons of continuous variables among three study groups. Time from diagnosis to (first) therapy (TTT) curves were built using the Kaplan–Meier method and compared by the (one-sided) log-rank test. Receiver operating characteristic (ROC) curve analysis was performed to determine the cutoff values of continuous variables that best distinguish disease progression. Multivariate analysis using the Cox regression model was performed to identify those variables independently associated with a greater/lower risk of disease progression (in terms of TTT) among M-CLL patients. All statistical analyses were performed with SPSS 25.0 (SPSS-IBM, Armonk, NY), and statistical significance was set at p-values ≤ 0.05, unless Bonferroni-adjusted p-values were applied (≤0.013).

## Results

### HCDR3 Hydropathy Index in CLL and Its Relationship With Other Disease Features

**Figure 1:**
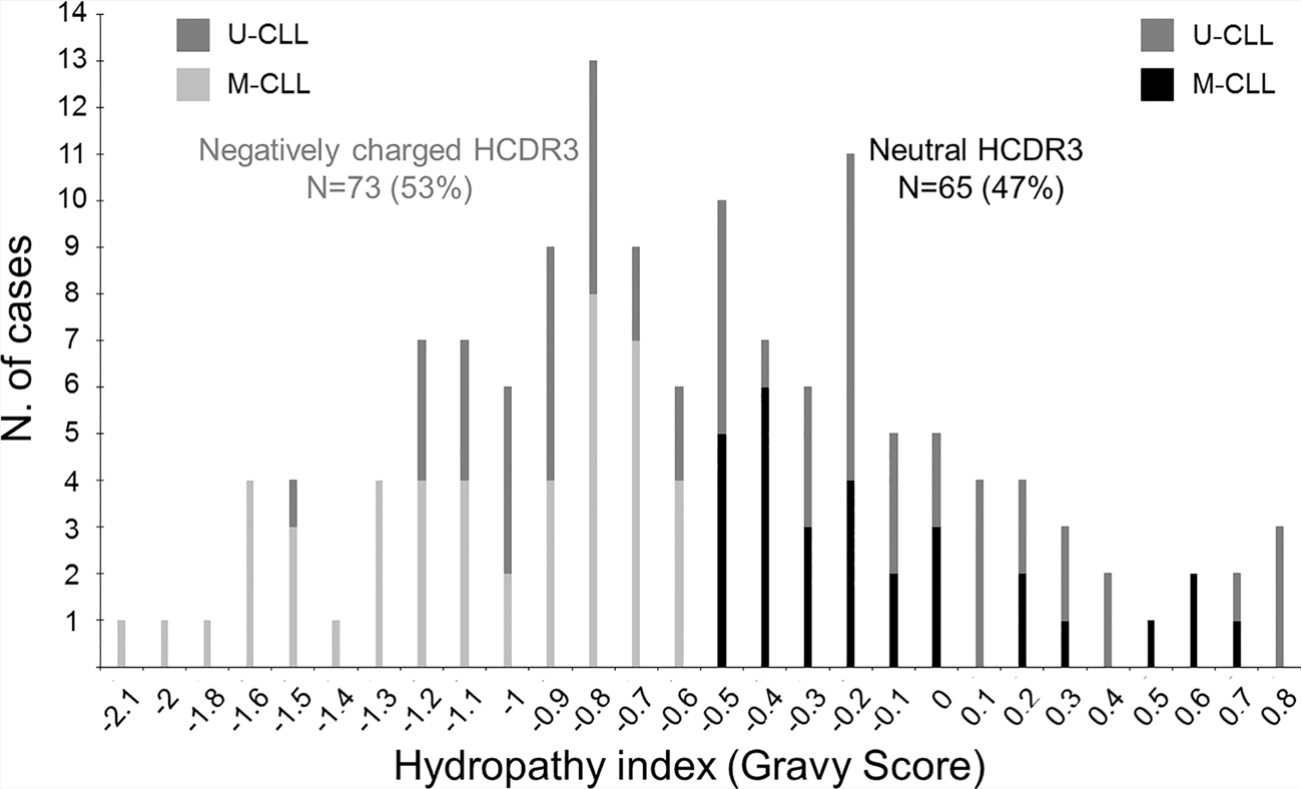
Distribution of CLL patients according to the hydropathy index—Gravy score (GS)—of the HCDR3 aa sequence of their BCR. Bars represent the number of CLL patients (N = 138) with different HCDR3 GS. Black bars correspond to M-CLL patients with a GS ≥ -0.5 (neutral HCDR3; N = 65), light gray bars correspond to M-CLL patients with GS < -0.5 (negatively charged HCDR3; N = 73), and dark gray bars represent cases with a ≥98% identity to the V(H) germline (U-CLL) independently of their GS.

Overall, a similar frequency of CLL patients with neutral (GS ≥ -0.5) and negatively charged (GS < -0.5) HCDR3 amino acid sequences was observed in our cohort: 65/138 (47%) *vs*. 73/138 (53%) CLL patients (p = 0.46), respectively (**Figure 1**). CLL patients with neutral or negatively charged HCDR3 showed no significant (p > 0.05) differences in age distribution, hemoglobin levels, leukocyte or platelet counts, CLL cell counts in blood, and the overall tumor cell cytogenetic profiles (**Table 1**).

**Figure 2:**
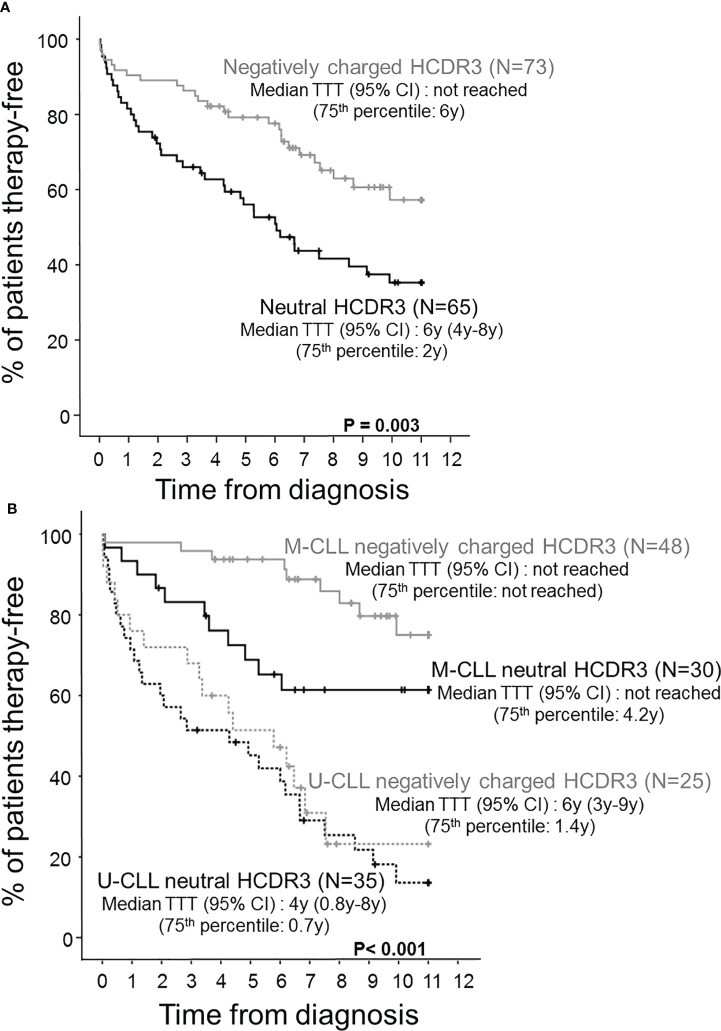
Time to therapy (TTT) survival curves of CLL patients distributed according to the hydropathy index of the HCDR3 aa sequence and the mutational status of their tumor cell BCR. Prognostic impact of the HCDR3 hydropathy index **(A)** and both the HCDR3 hydropathy index and the IGHV mutational status **(B)** on the outcome of CLL patients assessed by their survival from diagnosis to first therapy (TTT).

In contrast, CLL patients with neutral HCDR3 sequences showed a significant predominance of men *vs*. women (72% *vs*. 47%, p = 0.002), together with a lower percentage of Rai stage 0 (46% *vs*. 74%, p = 0.01) and Binet stage A cases (57% *vs*. 79%, p = 0.006), a higher proportion of cases with thrombocytopenia (21% of cases *vs*. 7%, p = 0.01), a lower proportion of M-CLL cases (46% *vs*. 66%, p = 0.02), and a lower median percentage of IGHV mutations (1% *vs*. 4%, p = 0.04) with longer HCDR3 sequences (median: 19 *vs*. 16 amino acids, p = 0.004) compared to CLL patients with negatively charged HCDR3 (**Table 1**). This CLL profile with neutral HCDR3 sequences translated into a significantly (p = 0.003) higher risk of disease progression (60% *vs*. 36%) and thereby also a higher percentage of cases that had required therapy at 2 y from diagnosis (31% *vs*. 11%, p = 0.004) (**Table 1**), and a significantly shortened TTT—median (95% confidence interval): 6 y (4–8 y) *vs*. not reached, p = 0.003 (**Figure 2A**). Of note, the prognostic impact of the HCDR3 hydropathy index was specifically restricted to M-CLL patients, while it did not show an impact on the already poorer outcome of U-CLL cases (**Figure 2B**).

### HCDR3 Hydropathy Index in M-CLL *vs*. U-CLL and Its Association With Other Disease Features

Based on the above findings, we subdivided M-CLL patients into cases with neutral HCDR3 (mean GS of -0.1) and patients with negatively charged HCDR3 sequences (mean GS of -1.1) and compared the features of these two subgroups of M-CLL *vs*. U-CLL cases (**Table 2**). Thus, Rai stage 0 (p = 0.007) predominated in the two M-CLL patient subgroups *vs*. U-CLL (**Table 2**). In contrast, greater median hemoglobin levels (150 *vs*. 130 g/L, p = 0.004) were found in M-CLL patients with a neutral HCDR3 (but not within those with a negatively charged HCDR3) *vs*. U-CLL. Overall, the number of PB leukocytes, total T cells, CD8^+^ T-cells, and tumor CLL cells, in blood, were all significantly increased in U-CLL compared with the two M-CLL patient groups, in the absence of significant differences between the later M-CLL groups (**Table 2**). Despite this, M-CLL with neutral HCDR3 sequences showed an intermediate frequency of cytogenetically altered cases between U-CLL and M-CLL with negatively charged HCDR3 (80% *vs*. 92% and 74%, respectively) together with a significantly greater proportion of del(17p)^+^ patients (11% *vs*. 0% and 2%, respectively; p = 0.02) (**Table 2**).

**Table 2:** Clinical and biological features of CLL patients classified according to their IGHV mutational status and the HCDR3 hydropathy index.

Patient features	U-CLL (N = 60)	M-CLL (N = 78)		p-value
		Neutral HCDR3 (N = 30)	Negatively charged HCDR3 (N = 48)	
Age at diagnosis (years)	64 (33–84)	61 (38–81)	63 (35–84)	0.21
Men/women^#^	38/22 (63%/37%)	20/10 (67%/33%)	23/25 (48%/52%)	0.16; 0.08*^b^* ^,^ *^c^*
Binet stage#				
A	37 (62%)	21 (70%)	37 (77%)	0.14
B	14 (23%)	2 (7%)	6 (13%)	
C	9 (15%)	7 (23%)	5 (10%)	
Rai stage^#^				
0	29/60 (48%)	19/30 (63%)	36/48 (75%)	0.007; 0.02*^a^*; 0.04*^b^*
I	6/60 (10%)	4/30 (13%)	3/48 (6%)	
II	12/60 (20%)	0/30 (0%)	2/48 (4%)	
III	6/60 (10%)	0/30 (0%)	2/48 (4%)	
IV	7/60 (12%)	7/30 (23%)	5/48 (10%)	
Hemoglobin (g/L)	130 (70–170)	150 (100–180)	130 (90–170)	0.010; 0.004*^a^*
Anemia (<100 g of hemoglobin/L)^#^	7/59 (12%)	0/30 (0%)	3/47 (6%)	0.12
N. of platelets (×10^9^/L)	149 (15–448)	158 (62–429)	164 (48–344)	0.63
Thrombocytopenia (<100 × 10^9^ platelets/L)^#^	7/58 (12%)	7/30 (23%)	5/47 (11%)	0.25
N. of PB leukocytes (×10^9^/L)	55 (10–576)	23 (6–245)	26 (8–241)	0.001; 0.010*^b^*
N. of PB total T-cells (×10^9^/L)	4 (0.5–14)	2.7 (0.9–7.4)	2.6 (0.6–9)	0.006; 0.003*^b^*
N. of PB CD4^+^ T-cells (×10^9^/L)	1.8 (0.6–6)	1.5 (0.5–4)	1.5 (0.3–4.3)	0.04
N. of PB CD8^+^ T-cells (×10^9^/L)	1.4 (0.1–8)	0.7 (0.2–5)	0.8 (0.1–5)	0.007; 0.005*^a^*
N. of PB monocytes (×10^9^/L)	0.7 (0.01–3)	0.5 (0.02–1.4)	0.4 (0.01–2)	0.12
N. of PB neutrophils (×10^9^/L)	6.3 (0.9–21)	6.3 (0.9–16)	6.0 (0.2–12)	0.47
N. of PB basophils (×10^9^/L)	0.07 (0.005–0.6)	0.06 (0.02–0.5)	0.06 (0.01–0.3)	0.66
N. of PB eosinophils (×10^9^/L)	0.2 (0.01–1.2)	0.2 (0.02–1.7)	0.2 (0.05–1)	0.66
N. of PB NK cells (×109/L)	0.7 (0.04–3)	0.6 (0.05–1.5)	0.4 (0.04–2.2)	0.014
Tumor B-cell clone size in blood (×10^9^/L)	47 (0.8–335)	16 (1.4–238)	17 (4.4–219)	0.001; 0.011*^b^*
% IGHV homology with germ line counterpart HCDR3 length of CLL clone (N. of aa)	99.6 (98.8–100) 21 (12–28)	93.7 (86–98) 16 (8–24)	93.3 (85–98) 16 (7–24)	<0.001; <0.001*^a^* ^,^ *^b^* <0.001;<0.001*^a^* ^,^ *^b^*
Cytogenetically altered CLL patients^#^	55/60 (92%)	24/30 (80%)	35/47 (74%)	0.05; 0.02*^b^*
Del(13q14)(*D13S25*)^#^	25/60 (42%)	11/30 (37%)	18/47 (38%)	0.88
Trisomy12^#^	17/60 (28%)	3/30 (10%)	7/47 (15%)	0.07; 0.04*^a^*
Del(11q)(*ATM*)^#^	9/60 (15%)	1/30 (3%)	2/47 (4%)	0.07; 0.06*^b^*
Del(17p)^#^	0/57 (0%)	3/28 (11%)	1/46 (2%)	0.02; 0.03*^a^*
N. of cytogenetically altered CLL cells (×10^9^/L)	28 (0–208)	8.6 (0–233)	6.3 (0–185)	0.12
Disease progression*^#^*	45/60 (75%)	11/30 (37%)	9/48 (19%)	<0.001; <0.001*^a^* ^,^ *^b^*
TTT < 2 years^#^	22/60 (37%)	5/30 (17%)	1/48 (2%)	<0.001; 0.04*^a^*; <0.001*^b^*; 0.03*^c^*
Deaths^#^	16/60 (27%)	2/30 (7%)	6/48 (12%)	0.03; 0.02*^a^*

Results expressed as median (range) or as # number of cases (percentage).

a U-CLL vs. M-CLL with neutral HCDR3.

b U-CLL vs. M-CLL with negatively charged HCDR3.

c M-CLL with neutral HCDR3 vs. M-CLL with negatively charged HCDR3.

CLL, chronic lymphocytic leukemia; M, mutated IGHV; N, number; PB, peripheral blood; TTT, time to therapy; U, unmutated IGHV.

Regarding outcome, M-CLL with neutral HCDR3 sequences showed an intermediate rate of disease progression (37%) compared to both U-CLL patients (75%) (p < 0.001) and M-CLL with negatively charged HCDR3 sequences (19%), after a similar median follow-up (**Table 2**). This was associated with a significantly lower percentage of M-CLL cases with negatively charged HCDR3 sequences that required therapy during the first 2 years after diagnosis (2%) compared to U-CLL (37%, p < 0.001) and M-CLL with a neutral HCDR3 (17%, p = 0.03) (**Table 2**). This translated into significantly prolonged TTT among M-CLL with negatively charged HCDR3 sequences compared to both M-CLL patients with a neutral HCDR3 sequence and U-CLL patients—75th percentile TTT (95% confidence interval): not reached *vs*. 4.2 and 0.9 y, respectively; p < 0.001) (**Figure 2B**).

Based on the results above, we specifically investigated the prognostic impact of the hydropathy index of the HCDR3 sequence of the tumor cell BCR compared to other clinical and laboratory variables in patients with M-CLL. Among all variables analyzed, Binet stage (p < 0.001), the number of total T-cells (p < 0.001), CD4^+^ T-cells (p = 0.002), CD8^+^ T-cells (p = 0.001), basophils (p = 0.04), the size of the tumor B-cell clone in blood (p = 0.003), del(11q) and/or del(17p) (p = 0.04) and the number of cytogenetically altered CLL cells (p = 0.001) in addition to the hydropathy index of the HCDR3 sequences of the tumor B-cell clone (p < 0.001) all showed a prognostic impact in the univariate analysis (**Table 3**). Multivariate analysis confirmed the independent adverse prognostic impact of neutral HCDR3 sequence of BCR (hazard ratio (HR), 12; 95% confidence interval (CI), 1.8 to 81; p = 0.01) together with an advanced Binet stage B/C (HR, 42.8; 95% CI, 1.7 to 1,073; p = 0.02) (**Table 3**).

**Table 3:** Univariate and multivariate analyses of prognostic factors with an impact on disease progression in M-CLL (N = 78).

Variables	N	Univariate analysis on disease progression		Multivariate analysis on disease progression	
		Median (95% CI) (y)	*p-value*	HR (95% CI)	*p-value*
Age at diagnosis					
≤ 65 y	50	NR	0.12		
> 65 y	28	NR			
Sex					
Men	43	NR	0.88		
Women	35	NR			
Binet stage					
A	58	NR			
B/C	20	8 (1.6–14.4)	<0.001	42.8 (1.7–1,073)	0.02
Anemia					
<100 g hemoglobin/L	3	9.9 (5–15.9)	0.27		
≥100 g hemoglobin/L	74	NR			
Thrombocytopenia					
<100 platelets × 10^9^/L	12	9.9	0.08		
≥100 platelets × 10^9^/L	65	NR			
N. of PB leukocytes (×10^9^/L)					
≤51.4	57	NR	0.06		
>51.4	20	NR			
N. of PB total T-cells (×10^9^/L)					
≤3.2	56	NR	<0.001		
>3.2	21	6.2 (2.7–9.7)			
N. of PB CD4^+^ T-cells (×10^9^/L)					
≤2.1	60	NR	0.002		
>2.1	15	8.7 (4.1–13.2)			
N. of PB CD8^+^ T-cells (×10^9^/L)					
≤1.7	63	NR	0.001		
>1.7	12	6.1 (5.9–6.4)			
N. of PB monocytes (×10^9^/L)					
≤1.04	67	NR	0.27		
>1.04	7	NR			
N. of PB neutrophils (×10^9^/L)					
≤11.4	73	NR	0.07		
>11.4	3	6.1			
N. of PB basophils (×10^9^/L)					
≤0.13	51	NR	0.04		
>0.13	5	8.7 (1.4–16)			
N. of PB eosinophils (×10^9^/L)					
≤0.12	24	NR	0.08		
>0.12	50	NR			
N. of PB NK cells (×10^9^/L)					
≤0.53	39	NR	0.31		
>0.53	32	NR			
Tumor B-cell clone size in blood (×10^9^/L)					
≤37.7	53	NR	0.003		
>37.7	23	9.93			
HCDR3 hydropathy index					
Neutral	30	4.2 (75^th^ percentile: NR)	<0.001	12 (1.8–81)	0.01
Negatively charged	48	NR (75^th^ percentile: NR)			
% IGHV homology					
≤96	65	NR	0.56		
>96	13	NR			
HCDR3 length of CLL clone					
≤21 aa	73	NR			
>21 aa	5	NR	0.86		
DH2 gene usage	22	NR	0.70		
JH6 gene usage	21	NR	0.86		
Del (11q) and/or del(17p)	7	4.2 (2.8–5.6)	0.04		
N. of cytogenetically altered CLL cells (×10^9^/L)					
≤33	57	NR			
>33	18	7.4 (2.2–12.5)	0.001		

aa, amino acids; CI, confidence interval; HR, hazard ratio; N, number of cases; NR, not reached; y, years.

### Distinctive Molecular Features of the BCR of M-CLL With Neutral *vs*. Negatively Charged HCDR3 Sequences

Interestingly, no significant differences were found between the two groups of M-CLL patients defined by having a neutral *vs*. negatively charged HCDR3, as regards the frequency of V(H) gene families used (**Table 4**). Despite this, both groups of M-CLL patients (with neutral and negatively charged HCDR3 sequences) more frequently used the VH3 gene at the expense of a lower frequency of VH1 gene usage compared to U-CLL patients—53% and 52% *vs*. 32%, (p = 0.05) and 17% and 8% *vs*. 47%, (p < 0.001), respectively (**Table 4**). In more detail, usage of the VH1-69 gene family was significantly associated with U-CLL—27% *vs*. 0% and 4%, p < 0.001—while VH4-34 was more frequently used in the two groups of (neutral and negatively charged HCDR3) M-CLL patients *vs*. U-CLL—17% and 21% *vs*. 3%, p = 0.02, respectively. Interestingly, VH3-7 was significantly associated with M-CLL with neutral HCDR3 sequences (17%) while rarely found in U-CLL (2%) (p = 0.01) (**Table 3**). Likewise, usage of the D(H)2 genes was more frequently observed in M-CLL with neutral HCDR3 sequences (43%) than in M-CLL with negatively charged HCDR3 (19%, p = 0.02) and U-CLL (22%, p = 0.03) patients (**Table 4**). Within the D(H)2 gene family, D(H)2-15 and D(H)2-21 were those family members more frequently expressed in M-CLL with neutral HCDR3 sequences *vs*. U-CLL (13% and 20% *vs*. 2% and 5%, respectively) (**Table 4**). In turn, U-CLL showed a higher frequency of D(H)3 than M-CLL patients (p = 0.001), the D(H)3-3 gene family mostly accounting for these differences as it was found in 30% of U-CLL *vs*. 7% of M-CLL with neutral HCDR3 and 12% of M-CLL with negatively charged HCDR3 sequences (p = 0.01) (**Table 4**).

**Table 4:** *IGHV(D)J* gene usage in CLL patients classified according to their IGHV mutational status and the HCDR3 hydropathy index.

BCR features	U-CLL (N = 60)	M-CLL (N = 78)		p-value
		Neutral HCDR3 (N = 30)	Negatively charged HCDR3 (N = 48)	
V(H) gene family usage^#^				
V1	28/60 (47%)	5/30 (17%)	4/48 (8%)	<0.001; 0.004*^a^* 0.000*^b^*
V1-69	16/60 (27%)	0/30 (0%)	2/48 (4%)	<0.001; 0.001*^a^ *^,^ *^b^*
V3	19/60 (32%)	16/30 (53%)	25/48 (52%)	0.05; 0.04*^a^*; 0.03*^b^*
V3-7	1/60 (2%)	5/30 (17%)	5/48 (10%)	0.03; 0.01*^a^*
V4	9/60 (15%)	8/30 (27%)	17/48 (35%)	0.05; 0.01*^b^*
V4-34	2/60 (3%)	5/30 (17%)	10/48 (21%)	0.02; 0.04*^a^*; 0.005*^b^*
D(H) gene family usage^#^				
D2	13/60 (22%)	13/30 (43%)	9/48 (19%)	0.04; 0.03*^a^*; 0.02*^c^*
D2-2	9/60 (15%)	3/30 (10%)	3/48 (6%)	0.34
D2-15	1/60 (2%)	4/30 (13%)	3/48 (6%)	0.08; 0.04*^a^*
D2-21	3/60 (5%)	6/30 (20%)	3/48 (6%)	0.04; 0.03*^a^*
D3	35/60 (58%)	6/30 (20%)	16/48 (33%)	0.001; 0.001*^a^*; 0.01*^b^*
D3-3	18/60 (30%)	2/30 (7%)	6/48 (12%)	0.01; 0.01*^a^*; 0.03*^b^*
J(H) gene family usage^#^				
J4	20/60 (33%)	7/30 (23%)	29/48 (60%)	0.002; 0.004*^b^*; 0.001*^c^*
J6	29/60 (48%)	11/30 (37%)	10/48 (21%)	0.01; 0.003*^b^*
Charge of HCDR3:				
J4 gene fraction	-0.167 (-0.296–0.029)	-0.076 (-0.325–0.115)	-0.209 (-0.946–0.115)	0.25
Non-J4 V(N1)D(N2) nucleotides	-0.392 (-1.150–0.447)	-0.107 (-0.346–0.670)	-0.936 (-1.702–-0.350)	<0.001; <0.001*^b^* ^,^ *^c^*
J6 gene fraction	-0.130 (-0.225–0.014)	-0.016 (-0.347–0.194)	-0.003 (-0.222–0.168)	0.010; 0.010*^b^*
Non-J6 V(N1)D(N2) nucleotides	-0.084 (-0.890–0.996)	-0.170 (-0.419–0.577)	-0.941 (-1.490–-0.572)	<0.001; <0.005*^b^* ^,^ *^c^*
D2 gene fraction	0.329 (-0.382–1.032)	0.010 (-0.286–0.698)	-0.258 (-0.596–0.029)	0.013; 0.003*^b^*
Non-D2 V(N1)(N2)J nucleotides	-0.502 (-1.380–-0.006)	-0.070 (-0.441–0.491)	-0.777 (-1.126–-0.471)	0.001; <0.010*^a^*; <0.001*^c^*
D3 gene fraction	-0.256 (-0.565–0.849)	-0.113 (-0.444–0.707)	-0.365 (-0.742–0.302)	0.10
Non-D3 V(N1)(N2)J nucleotides	-0.347 (-1.553–0.208)	0.007 (-0.570–0.351)	-0.712 (-1.057–-0.349)	0.001; <0.01*^b^*; 0.013*^c^*
HCDR3 length in patients aged ≤65y	21 (12–28)	14 (8–24)	16 (7–23)	0.001; 0.001*^a^*; 0.008*^b^*
HCDR3 length in patients aged >65y	20 (13–28)	18 (15–24)	15 (9–24)	<0.001; <0.001*^b^*; 0.013*^c^*
Stereotyped IGHV^#^	17/60 (28%)	5/30 (17%)	5/48 (10%)	0.06; 0.02*^b^*
Subsets (1,2,8)^#^	4/60 (7%)	2/30 (7%)	1/48 (2%)	0.50

Results expressed as median (range) or as # number of cases (percentage).

a U-CLL vs. M-CLL with neutral HCDR3.

b U-CLL vs. M-CLL with negatively charged HCDR3.

c M-CLL with neutral HCDR3 vs. M-CLL with negatively charged HCDR3.

CLL, chronic lymphocytic leukemia; M, mutated IGHV; N, number; U, unmutated IGHV.

Regarding J(H) gene usage, M-CLL with negatively charged HCDR3 sequences showed a significantly higher frequency of J(H)4 genes (60%) than both M-CLL with a neutral HCDR3 (23%) (p = 0.001) and U-CLL (33%) (p = 0.004) patients (**Table 4**). Likewise, a significantly lower percentage of M-CLL with negatively charged HCDR3 sequences showed J(H)6 gene usage (21%) compared to U-CLL (48%, p = 0.003) (**Table 4**). Interestingly, the lower use of JH6 and DH2 genes in M-CLL with negatively charged HCDR3 sequences was associated with different charges of the specific HCDR3 amino acids comprised by these coding genes *vs*. U-CLL patients (p = 0.010 and p = 0.003, respectively) (**Table 4**). In contrast, the charge of the HCDR3 amino acids comprised by JH4 and DH3 genes did not show differences between U-CLL and both M-CLL groups (**Table 4**). It should be noted, however, that the HCDR3 fraction comprised by nucleotides distinct to those included in the above referred JH and DH gene sequences showed always a significantly lower charge in M-CLL patients with negatively charged sequences compared to M-CLL with neutral HCDR3 sequences (**Table 4**).

As expected, U-CLL had longer HCDR3 (median: 21 amino acids) than M-CLL (median: 16 amino acids) (p < 0.001) (**Table 2**). However, since the length of the BCR HCDR3 sequences differs between B-cells from younger and older subjects (27), we grouped our patients into younger adults (≤65 y) and older (>65 y) patients. Interestingly, older M-CLL patients with a negatively charged HCDR3 showed shorter HCDR3 sequences (median: 15 amino acids) than M-CLL with neutral HCDR3 (median: 18 amino acids) (p = 0.013) (**Table 4**). Furthermore, older M-CLL patients with neutral HCDR3 had similarly longer HCDR3 sequences (median: 18 amino acids) to those of U-CLL patients (median: 20 amino acids) (p = 0.47) (**Table 4**). Finally, M-CLL with negatively charged HCDR3 sequences showed a significantly lower frequency of stereotyped IGHV sequences (10%) compared to U-CLL (28%) (p = 0.02) (**Table 4**), with a similarly low incidence of stereotyped IGHV sequences corresponding to the more aggressive (#1, #2, #8) CLL subsets (**Table 4**).

## Discussion

B-cells are a key component of the adaptive immune system (28). Their function is typically triggered through BCR-mediated recognition of specific antigens (28). Specific binding of BCR to antigens is mostly mediated through unique HCDR3 (and also LCDR3) regions capable of identifying and attaching to complementary epitopes in the recognized antigen (28). For adequate binding to the antigen, electrostatic links with the BCR are required (29). Thereby, the HCDR3 charge plays a critical role in antigen binding to the BCR and recognition by B-cells (30). Importantly, during antigen-driven maturation, B-cells modify their HCDR3 sequences to enhance their affinity for specific antigen triggers (30). This includes acquisition of somatic mutations involving the HCDR3 region, which progressively confer more negatively charged amino acid sequences for higher affinity antigen binding by both the BCR and the future B-cell derived (higher-affinity) antibodies (31). In addition, due to its key role in antigen recognition, the interaction of the BCR with the BM microenvironment also plays a critical role at an earlier stage, during lymphopoiesis, in selecting B-cell precursors that carry a functional BCR (31).

For decades now, studies have accumulated which support an important role for BCR-mediated expansion of tumor cells in CLL (32, 33) in the absence of a common genetic driver (6). Thus, CLL cells show biased usage of specific *IGHV(D)J* gene families, with overrepresentation of some genes such as *IGHV1-69*, *IGHV4-34*, and *IGHV3-21* (34). Of note, these genes are differentially distributed among the two major prognostic subgroups of CLL defined according to the mutational status of the BCR (U-CLL and M-CLL) (35). Accordingly, U-CLL cells have polyreactive BCRs that may respond to a wide spectrum of epitopes (36, 37), as typically required during selection of recently produced immature B-lymphocytes in BM (38), whereas M-CLL cells are more mature B-cells that have undergone somatic hypermutation, whose BCRs are (potentially) less responsive to external signals, while more specific for a given epitope (39–41). Among other factors, this might also contribute to explain the more aggressive clinical course (42, 43) and the shortened survival of U-CLL vs. M-CLL (44). As a consequence, the I*GHV* gene mutational status currently represents one of the most relevant prognostic determinants in CLL (44).

Similarly to normal B-cells (31), here we show that CLL cells also display a Gaussian distribution according to the hydropathy index of their BCR, slightly skewed toward negatively charged HCDR3 amino acid sequences. Interestingly, when we divided our patients into cases with neutral (mean GS of -0.1) vs. more negatively charged (mean GS of -1.1) HCDR3 sequences, two subgroups of CLL patients with clearly distinct clinical and biological features emerged. Thus, CLL patients with neutral HCDR3 sequences showed a clear predominance of men, U-CLL with longer HCDR3 sequences, a lower frequency of IGHV gene mutation, and higher frequency of more advanced stages of the disease, in association with a higher rate of disease progression and shorter TTT. Interestingly, shortening of HCDR3 sequences with a trend to negatively charged BCRs is a typical feature of selection of B-cell precursors in BM required for the survival of B-cells that will enter the mature B-cell repertoire (31). Based on these findings, our results suggest that expanded CLL cells in patients with neutral and longer HCDR3 sequences might reflect an earlier tumor cell origin in BM (45–47). Among other factors, this might also contribute to explain the greater frequency of more advanced stages of disease at diagnosis (48), together with an increased rate of disease progression vs. patients with negatively charged HCDR3 sequences. Nevertheless, these differences could be potentially due to the fact that CLL cases with neutral HCDR3 sequences included a higher fraction of U-CLL vs. M-CLL patients.

To investigate the potential independent value of both variables (the BCR mutational status and its hydropathy index), we separately studied the features of CLL patients with neutral vs. negatively charged HCDR3 sequences among U-CLL and M-CLL cases. Thus, U-CLL cases with a neutral and negatively charged HCDR3 showed similar clinical and biological features associated with a uniformly poorer outcome, in line with previous observations (49–51). In contrast, the HCDR3 hydropathy index identified two different prognostic subgroups of M-CLL. These included a subgroup of M-CLL with neutral HCDR3 who displayed intermediate clinical, genetic, and prognostic features between the classical U-CLL and M-CLL patients with a negatively charged BCR. Thus, M-CLL with neutral HCDR3 showed predominance of men—similar to that found in U-CLL, but with significantly higher hemoglobin levels—in association with a higher frequency of thrombocytopenia and an intermediate frequency of cytogenetically altered cases between U-CLL and the other M-CLL patients, at the expense of a greater frequency of del(17p). At present, it is well established that progression of MBL toward CLL is associated with a more prominent male predominance and greater frequency of U-CLL (52). Male predominance among M-CLL cases with neutral HCDR3 might also contribute to explain the greater hemoglobin levels observed in these patients, which contrasts with the higher frequency of thrombocytopenia compared to M-CLL with negatively charged HCDR3 sequences. This together with the greater frequency of more advanced stage of the disease among M-CLL with a neutral vs. negatively charged HCDR3 would support a poorer outcome within M-CLL for the former patient group, as confirmed here *via* an adverse impact on the time elapsed from diagnosis to first therapy among M-CLL patients with neutral vs. negatively charged HCDR3 sequences.

From the molecular point of view, M-CLL with neutral HCDR3 showed DJ footprints compatible with a more immature BCR repertoire associated with preferential usage of D(H)2 *IGHV* gene segments (53), in the absence of a biased use of JH4 gene segments, as found in M-CLL cases with negatively charged HCDR3 sequences, being biased use of JH4 gene segments a typical feature of more mature PB B lymphocytes (54). In addition, we also observed longer HCDR3 sequences in older (>65 y) patients who had U-CLL and M-CLL with neutral HCDR3 vs. M-CLL with negatively charged HCDR3 sequences, in line with what might be expected among older subjects (27). Despite U-CLL and M-CLL with neutral HCDR3 shared HCDR3 sequences which typically had no traits associated with a mature B-cell repertoire, important differences were still observed in the IGHV repertoire of CLL cells of both patient groups as regards the usage of the VH1 and VH3 gene segments, further emphasizing also the biological differences between them.

Altogether, our findings show that based on the HCDR3 hydropathy index of HCDR3 sequences, two clearly distinct subgroups of M-CLL patients with different clinical, genetic, and prognostic features can be identified which are characterized by neutral vs. negatively charged BCRs, associated with molecular features of precursor vs. peripheral/mature B-cells, respectively. Further studies are needed to elucidate the precise mechanisms involved in determining the role of these different BCR profiles (compared to other prognostic factors such as ZAP70) in the distinct clinical behavior and outcome of both groups of M-CLL patients and facilitate implementation of assays for routine assessment of the HCDR3 hydropathy index in M-CLL in the clinical settings.

## Data Availability Statement

The original contributions presented in the study are included in the article/**Supplementary Material**. Further inquiries can be directed to the corresponding authors.

## Ethics Statement

The studies involving human participants were reviewed and approved by the local Institutional Ethics Committee, University Hospital of Salamanca (code of approval: CEIC-PI4705/2017). The patients/participants provided their written informed consent to participate in this study.

## Author Contributions

AR-C, JA, and AO contributed to conception and design of the study. BF, GO, IC, and MA organized the database. CP contributed to bioinformatics calculations. MP and AG-M contributed to genomic collection, storage, and quality control. AG-M performed part of the statistical analysis and critical review of manuscript. MGD, FF and AS-R contributed in the clinical part of the manuscript and critical review of manuscript. AR-C wrote the first draft of the manuscript. All authors contributed to the manuscript revision and read and approved the submitted version.

## Funding

This work was supported by the following grants: FS/37-2017, from the Fundación Memoria D. Samuel Solórzano, Universidad de Salamanca; FIS PI17/00399-FEDER, from the Fondo de Investigación Sanitaria of Instituto de Salud Carlos III, Madrid, Spain; 0639_IDIAL_NET_3_E, from cooperative network EP-INTERREG V A España Portugal (POCTEP); and ECRIN-M3, Accelerator Award Full, Cancer Research UK, Fundación Científica de la Asociación Española Contra el Cáncer (AECC), Fondazione AIRC per la Ricerca sul Cancro. The funders had no role in the study design, data collection and analysis, decision to publish, or preparation of the manuscript.

## Conflict of Interest

The authors declare that the research was conducted in the absence of any commercial or financial relationships that could be construed as a potential conflict of interest.

## Publisher’s Note

All claims expressed in this article are solely those of the authors and do not necessarily represent those of their affiliated organizations, or those of the publisher, the editors and the reviewers. Any product that may be evaluated in this article, or claim that may be made by its manufacturer, is not guaranteed or endorsed by the publisher.

## Supplementary Material

Supplementary Figure 1 Kyte-Doolittle numerical scale of aminoacids (aa) and the formula to calculate the Gravy Score (GS) of HCDR3 (hydropathy index). The biochemical nature of aa according to their Kyte-Doolittle value and the algorithm used to calculate the GS of the HCDR3 sequence in our patients are shown in **(A, B)**, respectively. In **(C)**, four different HCDR3 sequences with their corresponding GS (where the sum of the Kyte-Doolittle values for individual aa divided by HCDR3 length provides the specific GS of individual sequences). As shown, different patients can share the same GS of their HCDR3, despite having different aa sequences.

## References

[1] Oppezzo P Dighiero G. Role of the B-Cell Receptor and the Microenvironment in Chronic Lymphocytic Leukemia. Blood Cancer J (2013) 3:e149. doi: 10.1038/bcj.2013.45 24056719 PMC3789209

[2] Hallek M. Chronic Lymphocytic Leukemia: 2020 Update on Diagnosis, Risk Stratification and Treatment. Am J Hematol (2019) 94(11):1266–87. doi: 10.1002/ajh.25595 31364186

[3] Hallek M Cheson BD Catovsky D Caligaris-Cappio F Dighiero G Döhner H. iwCLL Guidelines for Diagnosis, Indications for Treatment, Response Assessment, and Supportive Management of CLL. Blood (2018) 131(25):2745–60. doi: 10.1182/blood-2017-09-806398 29540348

[4] Dighiero G Hamblin TJ. Chronic Lymphocytic Leukemia. Lancet (2008) 371(9617):1017–29. doi: 10.1016/S0140-6736(08)60456-0 18358929

[5] International CLL-IPI working group. An International Prognostic Index for Patients With Chronic Lymphocytic Leukaemia (CLL-IPI): A Meta-Analysis of Individual Patient Data. Lancet Oncol (2016) 17(6):779–90. doi: 10.1016/S1470-2045(16)30029-8 27185642

[6] Puente XS Beá S Valdés-Mas R Villamor N Gutiérrez-Abril J Martín-Subero JI. Non-Coding Recurrent Mutations in Chronic Lymphocytic Leukemia. Nature (2015) 526(7574):519–24. doi: 10.1038/nature14666 26200345

[7] D´Avola A Drennan S Tracy I Henderson I Chiecchio L Larrayoz M. Surface IgM Expression and Function are Associated With Clinical Behavior, Genetic Abnormalities, and DNA Methylation in CLL. Blood (2016) 128(6):816–26. doi: 10.1182/blood-2016-03-707786 27301861

[8] Vardi A Agathangelidis A Sutton LA Ghia P Rosenquist R Stamatopoulos K. Immunogenetic Studies of Chronic Lymphocytic Leukemia: Revelations and Speculations About Ontogeny and Clinical Evolution. Cancer Res (2014) 74(16):4211–6. doi: 10.1158/0008-5472.CAN-14-0630 25074616

[9] Chiorazzi N Stevenson FK. Celebrating 20 Years of IGHV Mutation Analysis in CLL. Hemasphere (2020) 4(1):e334. doi: 10.1097/HS9.0000000000000334 32382709 PMC7000474

[10] Damle RN Ghiotto F Valetto A Albesiano E Fais F Yan XJ. B-Cell Chronic Lymphocytic Leukemia Cells Express a Surface Membrane Phenotype of Activated, Antigen-Experienced B Lymphocytes. Blood (2002) 99(11):4087–93. doi: 10.1182/blood.v99.11.4087 12010811

[11] Forconi F Potter KN Wheatley I Darzentas N Sozzi E Stamatopoulos K. The Normal IGHV1-69-Derived B-Cell Repertoire Contains Stereotypic Patterns Characteristic of Unmutated CLL. Blood (2010) 115(1):71–7. doi: 10.1182/blood-2009-06-225813 19887677

[12] Dono M Cerruti G Zupo S. The CD5^+^ B-Cell. Int J Biochem Cell Biol (2004) 36:2105–11. doi: 10.1016/j.biocel.2004.05.017 15313456

[13] Küppers R Zhao M Hansmann ML Rajewsky K. Tracing B Cell Development in Human Germinal Centres by Molecular Analysis of Single Cells Picked From Histological Sections. EMBO J (1993) 12(13):4955–67. doi: 10.1002/j.1460-2075.1993.tb06189.x PMC413756 8262038

[14] Chiorazzi N Ferrarini M. Cellular Origin(s) of Chronic Lymphocytic Leukemia: Cautionary Notes and Additional Considerations and Possibilities. Blood (2011) 117(6):1781–91. doi: 10.1182/blood-2010-07-155663 PMC3056635 21148333

[15] Agathangelidis A Darzentas N Hadzidimitriou A Brochet X Murray F Yan XJ. Stereotyped B-Cell Receptors in One-Third of Chronic Lymphocytic Leukemia: A Molecular Classification With Implications for Targeted Therapies. Blood (2012) 119(19):4467–75. doi: 10.1182/blood-2011-11-393694 PMC3392073 22415752

[16] Stamatopoulos K Agathangelidis A Rosenquist R Ghia P. Antigen Receptor Stereotypy in Chronic Lymphocytic Leukemia. Leukemia (2017) 31(2):282–91. doi: 10.1038/leu.2016.322 27811850

[17] Stamatopoulos K Belessi C Moreno C Boudjograh M Guida G Smilevska T. Over 20% of Patients With Chronic Lymphocytic Leukemia Carry Stereotyped Receptors: Pathogenic Implications and Clinical Correlations. Blood (2007) 109:259–70. doi: 10.1182/blood-2006-03-012948 16985177

[18] Dighiero G Oppezzo P. What do Somatic Hypermutation and Class Switch Recombination Teach Us About Chronic Lymphocytic Leukemia Pathogenesis? Curr Top Microbiol Immunol (2005) 294:71–89. doi: 10.1007/3-540-29933-5_5 16323428

[19] Ivanov I Link J Ippolito GC Schroeder HW Jr. “Constraints on Hydropathy and Sequence Composition of HCDR3 are Conserved Across Evolution”. In: Zanetti M, editor. The Antibodies. London: Taylor and Francis (2002). p. 43–67.

[20] Ivanov II Schelonka RL Zhuang Y Gartland GL Zemlin M Schroeder HW Jr. Development of the Expressed Ig CDR-H3 Repertoire is Marked by Focusing of Constraints in Length, Amino Acid Use, and Charge That Are First Established in Early B Cell Progenitors. J Immunol (2005) 174(12):7773–80. doi: 10.4049/jimmunol.174.12.7773 15944280

[21] Fais F Ghiotto F Hashimoto S Sellars B Valetto A Allen SL. Chronic Lymphocytic Leukemia B Cells Express Restricted Sets of Mutated and Unmutated Antigen Receptors. J Clin Invest (1998) 102:1515–25. doi: 10.1172/JCI3009 PMC509001 9788964

[22] Rosenquist R Ghia P Hadzidimitriou A Sutton LA Agathangelidis A Baliakas P. Immunoglobulin Gene Sequence Analysis in Chronic Lymphocytic Leukemia: Updated ERIC Recommendations. Leukemia (2017) 31(7):1477–81. doi: 10.1038/leu.2017.125 PMC5508071 28439111

[23] Henriques A Rodríguez-Caballero A Criado I Langerak AW Nieto WG Lécrevisse Q. Molecular and Cytogenetic Characterization of Expanded B-Cell Clones From Multiclonal Versus Monoclonal B-Cell Chronic Lymphoproliferative Disorders. Haematologica (2014) 99(5):897–907. doi: 10.3324/haematol.2013.098913 24488564 PMC4008118

[24] Criado I Muñoz-Criado S Rodríguez-Caballero A Nieto WG Romero A Fernández-Navarro P. Host Virus and Pneumococcus-Specific Immune Responses in High-Count Monoclonal B-Cell Lymphocytosis and Chronic Lymphocytic Leukemia: Implications for Disease Progression. Haematologica (2017) 102(7):1238–46. doi: 10.3324/haematol.2016.159012 PMC5566034 28385786

[25] Kyte J Doolittle RF. A Simple Method for Displaying the Hydropathic Character of a Protein. J Mol Biol (1982) 157(1):105–32. doi: 10.1016/0022-2836(82)90515-0 7108955

[26] Criado I Rodríguez-Caballero A Gutiérrez ML Pedreira CE Alcoceba M Nieto W. Low-Count Monoclonal B-Cell Lymphocytosis Persists After Seven Years of Follow Up and is Associated With a Poorer Outcome. Haematologica (2018) 103(7):1198–208. doi: 10.3324/haematol.2017.183954 PMC6029554 29567775

[27] Pickman Y Dunn-Walters D Mehr R. BCR CDR3 Length Distributions Differ Between Blood and Spleen and Between Old and Young Patients, and TCR Distributions can be Used to Detect Myelodysplastic Syndrome. Phys Biol (2013) 10(5):56001. doi: 10.1088/1478-3975/10/5/056001 23965732

[28] Kwak K Akkaya M Pierce SK. B Cell Signaling in Context. Nat Immunol (2019) 20(8):963–9. doi: 10.1038/s41590-019-0427-9 31285625

[29] DeKosky BJ Lungu OI Park D Johnson EL Charab W Chrysostomou C. Large-Scale Sequence and Structural Comparisons of Human Naïve and Antigen-Experienced Antibody Repertoires. Proc Natl Acad Sci USA (2016) 113(19):E2636–45. doi: 10.1073/pnas.1525510113 PMC4868480 27114511

[30] Khass M Vale AM Burrows PD Schroeder HW Jr. The Sequences Encoded by Immunoglobulin Diversity (DH) Gene Segments Play Key Roles in Controlling B-Cell Development, Antigen-Binding Site Diversity, and Antibody Production. Immunol Rev (2018) 284(1):106–19. doi: 10.1111/imr.12669 29944758

[31] Kaplinsky J Li A Sun A Coffre M Koralov SB Arnaout R. Antibody Repertoire Deep Sequencing Reveals Antigen-Independent Selection in Maturing B Cells. Proc Natl Acad Sci USA (2014) 111(25):E2622–9. doi: 10.1073/pnas.1403278111 PMC4078805 24927543

[32] Haselager MV Kater AP Eldering E. Proliferative Signals in Chronic Lymphocytic Leukemia; What Are We Missing? Front Oncol (2020) 10:592205. doi: 10.3389/fonc.2020.592205 33134182 PMC7578574

[33] Greaves M. Clonal Expansion in B-CLL: Fungal Drivers or Self-Service? J Exp Med (2013) 210(1):1–3. doi: 10.1084/jem.20122739 23319726 PMC3549708

[34] Hengeveld PJ Levin MD Martijn Kolijn P Langerak AW. Reading the B-Cell Receptor Immunome in Chronic Lymphocytic Leukemia: Revelations and Applications. Exp Hematol (2021) 93:14–24. doi: 10.1016/j.exphem.2020.09.194 32976948

[35] Mauerer K Zahrieh D Gorgun G Li A Zhou J Ansén S. Immunoglobulin Gene Segment Usage, Location and Immunogenicity in Mutated and Unmutated Chronic Lymphocytic Leukemia. Br J Haematol (2005) 129(4):499–510. doi: 10.1111/j.1365-2141.2005.05480.x 15877732

[36] Hwang HK Trama AM Kozink DM Chen X Wiehe K Cooper AJ. IGHV1-69 B Cell Chronic Lymphocytic Leukemia Antibodies Cross-React With HIV-1 and Hepatitis C Virus Antigens as Well as Intestinal Commensal Bacteria. PLoS One (2014) 9(3):e90725. doi: 10.1371/journal.pone.0090725 24614505 PMC3948690

[37] Hatzi K Catera R Moreno Atanasio C Fischetti VA Allen SL Kolitz JE. Chronic Lymphocytic Leukemia Immunoglobulins Display Bacterial Reactivity That Converges and Diverges From Auto-/Poly-Reactivity and IGHV Mutation Status. Clin Immunol (2016) 172:44–51. doi: 10.1016/j.clim.2016.08.020 27586592

[38] Cariappa A Chase C Liu H Russell P Pillai S. Naïve Recirculating B Cells Mature Simultaneously in the Spleen and Bone Marrow. Blood (2007) 109(6):2339–45. doi: 10.1182/blood-2006-05-021089 17119110

[39] Burger JA Chiorazzi N. B Cell Receptor Signaling in Chronic Lymphocytic Leukemia. Trends Immunol (2013) 34(12):592–601. doi: 10.1016/j.it.2013.07.002 23928062 PMC3898793

[40] Herve M Xu K Ng YS Wardemann H Albesiano E Messmer BT. Unmutated and Mutated Chronic Lymphocytic Leukemias Derive From Self-Reactive B Cell Precursors Despite Expressing Different Antibody Reactivity. J Clin Invest (2005) 115(6):1636–43. doi: 10.1172/JCI24387 PMC1088018 15902303

[41] ten Hacken E Burger JA. Molecular Pathways: Targeting the Microenvironment in Chronic Lymphocytic Leukemia—Focus on the B-Cell Receptor. Clin Cancer Res (2014) 20(3):548–56. doi: 10.1158/1078-0432.CCR-13-0226 24323900

[42] Damle RN Wasil T Fais F Ghiotto F Valetto A Allen SL. Ig V Gene Mutation Status and CD38 Expression as Novel Prognostic Indicators in Chronic Lymphocytic Leukemia. Blood (1999) 94(6):1840–7. doi: 10.1182/blood.V94.6.1840 10477712

[43] Hamblin TJ Davis Z Gardiner A Oscier DG Stevenson FK. Unmutated Ig VH Genes are Associated With a More Aggressive Form of Chronic Lymphocytic Leukemia. Blood (1999) 94:1848–54. doi: 10.1182/blood.V94.6.1848 10477713

[44] Chiorazzi N Chen S-S Rai KR. Chronic Lymphocytic Leukemia. Cold Spring Harb Perspect Med (2021) 11(2):a035220. doi: 10.1101/cshperspect.a035220 32229611 PMC7849345

[45] Alizadeh AA Majeti R. Surprise! HSC Are Aberrant in Chronic Lymphocytic Leukemia. Cancer Cell (2011) 20(2):135–6. doi: 10.1016/j.ccr.2011.08.001 21840478

[46] Kikushige Y Ishikawa F Miyamoto T Shima T Urata S Yoshimoto G. Self-Renewing Hematopoietic Stem Cells Is the Primary Target in Pathogenesis of Human Chronic Lymphocytic Leukemia. Cancer Cell (2011) 20(2):246–59. doi: 10.1016/j.ccr.2011.06.029 21840488

[47] Rodríguez-Caballero A Henriques A Criado I Langerak AW Matarraz S López A. Subjects With Chronic Lymphocytic Leukemia-Like B Cell Clones With Stereotyped B-Cell Receptors Frequently Show MDS-Associated Phenotypes on Myeloid Cells. Br J Haematol (2015) 168(2):258–67. doi: 10.1111/bjh.13127 25252186

[48] Pasikowska M Walsby E Apollonio B Cuthill K Philips E Coulter E. Phenotype and Immune Function of Lymph Node and Peripheral Blood CLL Cells are Linked to Transendothelial Migration. Blood (2016) 128(4):563–73. doi: 10.1182/blood-2016-01-683128 27252234

[49] Görgün G Holderried TAW Zahrieh D Neuberg D Gribben JG. Chronic Lymphocytic Leukemia Cells Induce Changes in Gene Expression of CD4 and CD8 T Cells. J Clin Invest (2005) 115:1797–805. doi: 10.1172/JCI24176 PMC1150284 15965501

[50] van Attekum MH Eldering E Kater AP. Chronic Lymphocytic Leukemia Cells are Active Participants in Microenvironmental Cross-Talk. Haematologica (2017) 102(9):1469–76. doi: 10.3324/haematol.2016.142679 PMC5685246 28775118

[51] Huergo-Zapico L Acebes-Huerta A González-Rodríguez AP Contesti J Gonzalez-García E Payer AR. Expansion of NK Cells and Reduction of NKG2D Expression in Chronic Lymphocytic Leukemia. Correlation With Progressive Disease. PLoS One (2014) 9(10):e108326. doi: 10.1371/journal.pone.0108326 25286418 PMC4186792

[52] Catovsky D Wade R Else M. The Clinical Significance of Patients´ Sex in Chronic Lymphocytic Leukemia. Haematologica (2014) 99(6):1088–94. doi: 10.3324/haematol.2013.101378 PMC4040913 24658818

[53] Mroczek ES Ippolito GC Rogosch T Hon Hoi K Hwangpo TA Brand MG. Differences in the Composition of the Human Antibody Repertoire by B Cell Subsets in the Blood. Front Immunol (2014) 5:96. doi: 10.3389/fimmu.2014.00096 24678310 PMC3958703

[54] Huang C Stewart AK Schwartz RS Stollar BD. Immunoglobulin Heavy Chain Gene Expression in Peripheral Blood B Lymphocytes. J Clin Invest (1992) 89(4):1331–43. doi: 10.1172/JCI115719 PMC442995 1556192

