# Supplementary figures and images for: The Hydropathy Index of the HCDR3 Region of the B-Cell Receptor Identifies Two Subgroups of IGHV-Mutated Chronic Lymphocytic Leukemia Patients With Distinct Outcome
Source: Front Oncol. 2021 Oct 26;11:723722. doi: 10.3389/fonc.2021.723722 (PMC8577851; doi:10.3389/fonc.2021.723722)

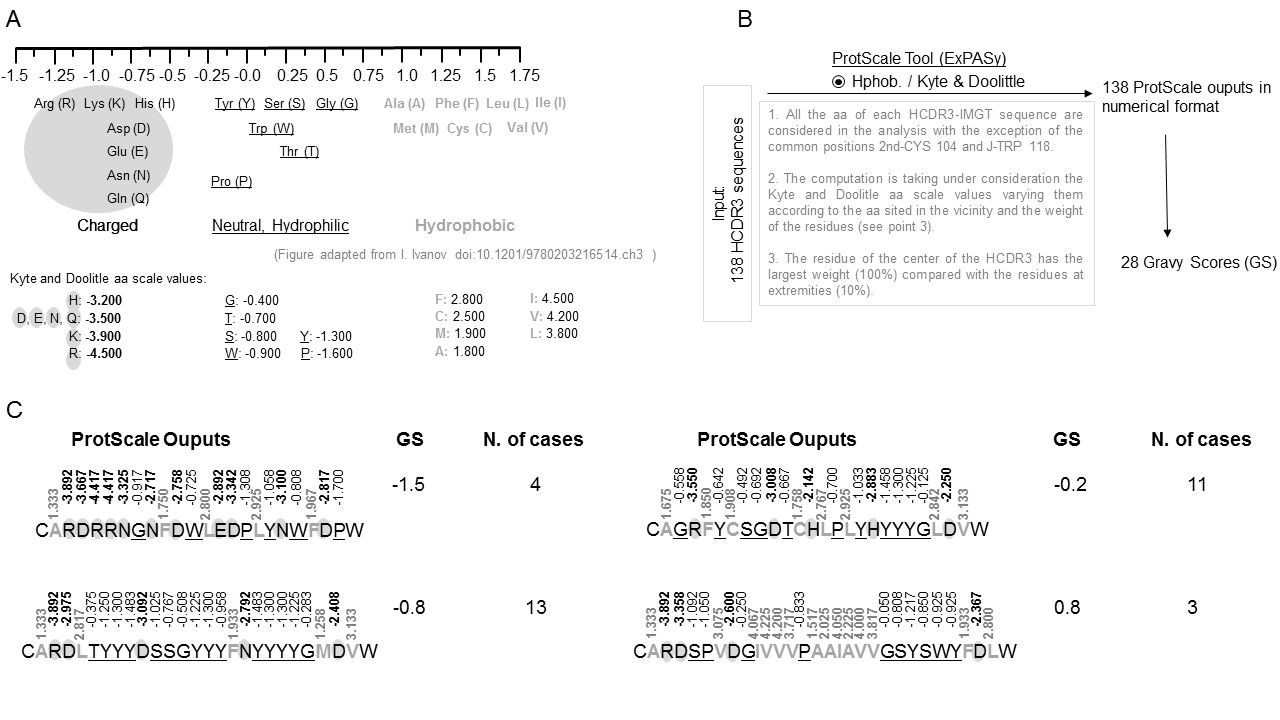

Supplement: Supplementary Figure 1 — Kyte-Doolittle numerical scale of aminoacids (aa) and the formula to calculate the Gravy Score (GS) of HCDR3 (hydropathy index). The biochemical nature of aa according to their Kyte-Doolittle value and the algorithm used to calculate the GS of the HCDR3 sequence in our patients are shown in (A, B), respectively. In (C), four different HCDR3 sequences with their corresponding GS (where the sum of the Kyte-Doolittle values for individual aa divided by HCDR3 length provides the specific GS of individual sequences). As shown, different patients can share the same GS of their HCDR3, despite having different aa sequences. [file Image_1.tif]
