# Supplementary material for: The Hydropathy Index of the HCDR3 Region of the B-Cell Receptor Identifies Two Subgroups of IGHV-Mutated Chronic Lymphocytic Leukemia Patients With Distinct Outcome
Source: Front Oncol. 2021 Oct 26;11:723722. doi: 10.3389/fonc.2021.723722 (PMC8577851; doi:10.3389/fonc.2021.723722)
Supplement: Supplementary file 3 [file Table_2.docx]

**SupplementaryTable 2.** *IGHV(D)J* genes used at low frequency among CLL patients classified according to their IGHV mutational status and the HCDR3 hydropathy index.

|  | **U-CLL (N=60)** | **M-CLL (N=78)** | | ***P-value*** |
| --- | --- | --- | --- | --- |
| **BCR features** |  | **Neutral CDR3**  **(N=30)** | **Negatively charged HCDR3**  **(N=48)** |  |
| V(H) family genes usage^#^  V2  V5  V6  V7 | 0/60 (0%)  3/60 (5%)  0/60 (0%)  1/60 (2%) | 1/30 (3%)  0/30 (0%)  0/30 (0%)  0/30 (0%) | 2/48 (4%)  0/48 (0%)  1/48 (2%)  0/48 (0%) | 0.30  0.14  0.39  0.52 |
| D(H) family genes usage^#^  D1  D1-1  D1-14 ORF  D1-26  D1-7  D4  D4-11 ORF  D4-17  D4-23 ORF  D5  D5-12  D5-18  D5-24 ORF  D6  D6-13  D6-19  D6-6 | 2/60 (3%)  0/60 (0%)  0/60 (0%)  1/60 (2%)  1/60 (2%)  1/60 (2%)  0/60 (0%)  1/60 (2%)  0/60 (0%)  1/60 (2%)  1/60 (2%)  0/60 (0%)  0/60 (0%)  8/60 (13%)  0/60 (0%)  7/60 (12%)  1/60 (2%) | 1/30 (3%)  1/30 (3%)  0/30 (0%)  0/30 (0%)  0/30 (0%)  1/30 (3%)  0/30 (0%)  0/30 (0%)  1/30 (3%)  4/30 (13%)  1/30 (3%)  2/30 (7%)  1/30 (3%)  4/30 (13%)  2/30 (7%)  2/30 (7%)  0/30 (0%) | 4/48 (8%)  0/48 (0%)  1/48 (2%)  3/48 (6%)  0/48 (0%)  5/48 (10%)  1/48 (2%)  1/48 (2%)  3/48 (6%)  3/48 (6%)  1/48 (2%)  0/48 (0%)  2/48 (4%)  11/48 (23%)  6/48 (13%)  5/48 (10%)  0/48 (0%) | 0.44  0.12  0.11  0.48  0.08; 0.02^a^  0.35  0.35  0.11 |
| J(H) family genes usage^#^  J3  J5  J2  J1 | 5/60 (8%)  4/60 (7%)  2/60 (3%)  0/60 (0%) | 6/30 (20%)  4/30 (13%)  1/30 (3%)  1/30 (3%) | 4/48 (8%)  4/48 (8%)  1/48 (2%)  0/48 (0%) | 0.19  0.57  0.92  0.16 |

Results expressed as # number of cases (percentage). ^a^ U-CLL vs M-CLL with neutral HCDR3, ^b^ U-CLL vs M-CLL with negatively charged HCDR3, ^c^ M-CLL with neutral HCDR3 vs M-CLL with negatively charged HCDR3.

Abbreviations (alphabetical order): **BCR:** B-cell receptor; **CLL:** chronic lymphocytic leukemia; **M:** mutated *IGHV*; **N:** Number; **U:** unmutated *IGHV*.
